# Surgical treatment of central grade 1 chondrosarcoma of the appendicular skeleton

**DOI:** 10.1007/s10195-013-0230-6

**Published:** 2013-03-06

**Authors:** Domenico Andrea Campanacci, Guido Scoccianti, Alessandro Franchi, Giuliana Roselli, Giovanni Beltrami, Massimiliano Ippolito, Giuseppe Caff, Filippo Frenos, Rodolfo Capanna

**Affiliations:** 1 Department of Orthopaedic Oncology and Reconstructive Surgery, Azienda Ospedaliera Universitaria Careggi, Florence, Italy; 2 Department of Pathology, University of Florence, Florence, Italy; 3 Department of Radiology, Azienda Ospedaliera Universitaria Careggi, Florence, Italy

**Keywords:** Chondrosarcoma, Bone sarcoma, Bone tumors, Orthopedic oncology

## Abstract

**Background:**

Diagnosis and treatment of low-grade chondrosarcoma remain controversial. We performed a review of a single-center series with the aims of assessing the oncologic outcome of these patients, verifying if intralesional curettage can be adequate treatment, and defining clinical criteria to support the surgeon and the oncologist in decision-making for surgery and subsequent follow-up.

**Materials and methods:**

A retrospective review of 85 patients was performed (61 females and 24 males, age range 20–76 years). The site of the lesion was the femur in 35 cases, humerus in 33, tibia in 15, and fibula in 2. Sixty-four patients were treated by intralesional curettage. Twenty-one patients with aggressive radiological patterns were treated with wide resection.

**Results:**

Mean follow-up was 67 months (range 24–206 months). Two patients developed local recurrence, both after intralesional curettage. The difference in incidence of recurrence was not statistically significant between the two groups. No distant metastases were observed. Postsurgical complications were significantly higher in the resection group.

**Conclusions:**

Low-grade chondrosarcoma of the appendicular skeleton without aggressive radiological patterns can be treated with intralesional surgery with good oncological outcome and very low rate of postsurgical complications. Wide resection, following surgical principles of malignant bone tumors, should be considered only when aggressive biologic behavior is evident on imaging.

## Introduction

Chondrosarcoma (CS) is the second most frequent primary malignant bone tumor after osteosarcoma. Central CS may grow primarily in the medullary canal of healthy bone or may be secondary to pre-existing benign enchondroma [1]. The prognosis of central CS is directly correlated with the histological grade of malignancy, which is assessed following the criteria described by Evans et al., considering cellularity, tumor matrix characteristics, nuclear features, and mitotic rate [2]. Histologic evaluation of cartilaginous tumors represents a challenging task for the pathologist, and consistent interobserver variability, concerning grading and the distinction between benign and malignant lesions, was observed [3, 4]. Moreover, in the past the concept of “borderline” lesion was introduced, with the aim of indicating a cartilaginous lesion more active than a benign enchondroma but less atypical than a grade 1 chondrosarcoma [5]. Other definitions were used to indicate borderline lesions, such as “atypical enchondroma,” “grade 0 chondrosarcoma,” and “cartilaginous lesion with unknown malignant potential” (CLUMP) [6, 7], but finally there was no common agreement on the adequacy and usefulness of these definitions, which have now generally been abandoned.

In cartilaginous tumors, biopsy can confirm the diagnostic hypothesis of cartilaginous lesion without being reliable in grading the tumor, since different areas with progressive grade of malignancy can be found within the same lesion and benign enchondroma may coexist with an area of dedifferentiation in a highly malignant tumor. For this reason, clinical and imaging criteria must be considered in decision-making regarding the surgical approach to low-grade chondrosarcoma.

Cartilaginous tumors of the appendicular skeleton have shown less aggressive biologic behavior than axial tumors [8–11], and good local control of the tumor with excellent long-term survival was obtained with both intralesional surgery and segmental resection in grade 1 CS of the appendicular skeleton [11–17]. Nevertheless, local recurrence of grade 1 CS may present a progression of malignancy with an influence on patient survival [18]. In recent decades, intralesional curettage with local adjuvants and bone filling with either bone grafts or cement has been widely used as surgical treatment of grade 1 CS of the appendicular skeleton [12, 15–17, 19–22]. On the other hand, in case of an aggressive aspect on imaging and cortical disruption of grade 1 CS, resection with wide margins, following the surgical criteria of primary bone sarcoma, has been recommended [5, 6, 13, 14, 23, 24]. We reviewed our experience of surgical treatment of central grade 1 CS of the appendicular skeleton with the aim of assessing the oncologic outcome of these patients, verifying if intralesional curettage can be adequate treatment, and defining clinical criteria to support the surgeon and oncologist in decision-making for surgery and subsequent follow-up.

## Materials and methods

A retrospective review of patients treated in authors’ institution for central grade 1 chondrosarcoma of long bones from 1994 to 2010 was performed. Ninety-nine patients meeting the above-mentioned criteria were identified. Fourteen patients were excluded from the present study due to follow-up of less than 24 months or insufficient clinical or radiological data. Eighty-five patients remained for evaluation. There were 61 females and 24 males with mean age of 50 years (range 20–76 years). The site of the lesion was the femur in 35 cases, humerus in 33, tibia in 15, and fibula in 2 cases.

All patients with definitive histological diagnosis of central grade 1 CS on analysis of the entire specimen were included in the present study. Histological diagnosis of grade 1 CS was done according to established criteria reported in the literature [3, 4, 25].

The need for informed consent was waived by the ethical committee since rights and interests of the patients would not be violated and their privacy and anonymity would be ensured by the study design. The research conformed to the Declaration of Helsinki.

**Fig. 1:**
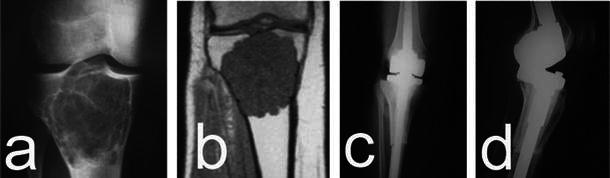
**a**, **b** Grade 1 CS of the proximal tibia involving the whole metaepiphysis. The anteroposterior (AP) radiographic view and magnetic resonance imaging (MRI) show the endosteal scalloping and cortical thinning consistent with aggressive imaging features. **c**, **d** Proximal tibia resection with wide margins and allograft–prosthesis composite reconstruction was performed

**Fig. 2:**
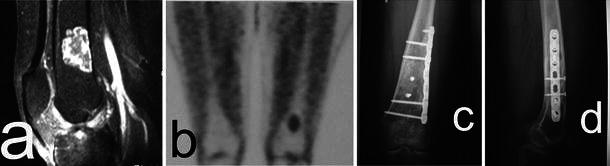
**a** Grade 1 CS of the distal femur. MRI lateral view. **b** Positive positron emission tomography (PET) scan. **c**, **d** AP and lateral (LL) radiographs at 5.5 years from surgery. Curettage, filling with allografts, and plate and screws fixation were performed

Imaging features of the lesion were considered in surgical treatment planning. Evidence of bone enlargement with cortical thinning or thickening and/or interruption, periosteal reaction, or presence of soft tissue mass led us to choose segmental resection with tumor-free margins (Fig. 1). In absence of the above-mentioned criteria of aggressiveness, in case of intraosseous cartilaginous lesion with mild or moderate endosteal cortical scalloping, intralesional curettage was performed (Fig. 2).

In 64 cases (75.3 %) intralesional curettage was performed using phenol and alcohol as local adjuvants in 69 % of cases. The defect was filled with allogenic bone chips in 60 cases; bone cement was employed to fill the defect in 3 cases, while in 1 patient a bone substitute was used. Prophylactic internal fixation was performed with plate in 16 patients.

Twenty-one patients (24.7 %) underwent segmental resection with wide surgical margins. Reconstruction was performed using a modular endoprosthesis in six cases, massive allograft in four cases (allograft alone in one case, osteoarticular allograft + vascularized fibular autograft in two cases, intercalary allograft + vascularized fibular autograft in one case), and an allograft–prosthetic composite in five cases (in one of these patients, total tibia resection was performed since he had been treated in another hospital with intramedullary locked nail fixation for a pathological fracture on a central grade 1 CS of the tibia with consequent contamination of the entire tibia; in this patient a long-stem rotating-hinge prosthesis was cemented into a total tibia massive allograft). In three patients, the resected intercalary segment was curetted on a separate table, sterilized by autoclave, and reimplanted combined with a vascularized fibular autograft in two cases and filled with cement in another patient. A vascularized fibular autograft alone was employed in two cases. In one case no reconstruction was performed (fibula).

All patients were clinically and radiologically evaluated at last follow-up, except for five patients who had been followed up by local orthopedic surgeons and did not come back to our unit. These patients were interviewed by phone, and the latest radiographic evaluation was adopted for oncological status and date of latest follow-up.

## Results

At average follow-up of 67 months (range 24–206 months), two patients had developed local recurrence and no distant metastases were observed. One of the local recurrences occurred in a 71-year-old man after intralesional curettage of a grade 1 CS of the distal tibia. The patient was temporarily lost to follow-up but after 3 years from surgery he came back with extensive local recurrence involving the distal third of the tibia and presenting soft tissue extension. The patient refused the proposed below-knee amputation and underwent distal tibia resection and reconstruction with ankle arthrodesis using allogenic cortical struts, bone autograft from iliac crest, and intramedullary anterograde locked nail fixation. Final histologic response on the resected specimen demonstrated marginal surgical margins and progression of malignancy in grade 2 CS. The patient required a secondary soleal rotational flap coverage for wound dehiscence and eventually developed a deep infection, finally treated by below-knee amputation. The second recurrence occurred 2 years after curettage and bone grafting of a lesion involving proximal fibula; the patient was treated with proximal fibula resection at another institution, and grade 1 was confirmed at histologic examination.

Both recurrences occurred after curettage (3.1 % of cases treated with this method); no recurrences were observed after resection. The difference between the two groups was not statistically significant according to Fisher’s exact test (*P* = 1.0).

After intralesional curettage (64 patients) a pathologic fracture of the femur was observed in 1 case (1.6 %). The fracture healed after conservative treatment.

Segmental resection was performed in 21 patients, and in 6 cases (28.6 %) a complication was observed, requiring surgical revision in 5 cases. In one patient with distal femur modular prosthesis, stem breakage occurred and conversion to total femur prosthesis was performed. Dislocation of an allograft reverse-prosthetic composite of the proximal humerus was observed in one case. One patient with an allograft–prosthetic composite of the proximal femur developed allograft resorption and acetabular erosion due to the bipolar prosthetic head; the allograft was removed and replaced with a modular prosthesis, and an acetabular cemented component was implanted. Two patients, treated with osteoarticular allograft of the proximal humerus associated with vascularized fibular autograft, sustained fracture of the allograft with collapse of the articular surface; the allograft was removed in both cases and replaced by a modular prosthesis in one case and by an allograft reverse-prosthetic composite in one case. In one case, stress fracture of the vascularized fibular graft after intercalary resection of the humerus was seen to heal spontaneously with conservative treatment.

No deep infections occurred besides the above-mentioned case after second surgery.

The incidence of complications was significantly higher in the resection group (Fisher’s exact test: 0.0007).

## Discussion

Surgical treatment of low-grade central CS has been a subject of debate for a long time. Some authors advocate wide resection margins, while others consider intralesional curettage sufficient for adequate local control [11–24]. This discordance is probably favored by two factors: interobserver variability in histologic diagnosis of cartilaginous tumors (overgrading of atypical enchondroma and undergrading of grade 2 chondrosarcoma) and inclusion of both appendicular and axial skeleton lesions in some of the reported series.

Central CS of the axial skeleton showed more aggressive biologic behavior than appendicular tumors, and high incidence of local recurrence after intralesional excision of grade 1 CS of the axial skeleton was reported [5, 9, 10, 24]. Conversely, in the appendicular skeleton, excellent local control and survival were reported after intralesional curettage of grade 1 CS [12, 15–17, 19–21]. Therefore, axial and appendicular lesions should be evaluated separately, as in this study.

The variability in histological grading and the consequent bias, which impacts on every work on this subject, is more difficult to overcome. Different imaging techniques or clinical patterns have been tested and proposed to support the diagnosis, but no conclusive data are currently available. In fact, a variable spectrum of imaging patterns can be associated with grade 1 CS, ranging from apparently benign asymptomatic enchondroma to clearly aggressive symptomatic malignant cartilaginous lesion. Lesion dimensions were argued to be indicative of malignancy, but this was not confirmed by recent investigations [14]. Cortical bone alterations such as endosteal scalloping, thinning, thickening, bone enlargement, and cortical interruptions or periosteal reaction were considered signs of malignancy, and computed tomography (CT) scan is the best option to evaluate cortical bone modifications. Bone enlargement and cortical thinning were found to be significant signs of malignancy in a recent report, and the authors suggested wide resection as surgical treatment when these imaging features are observed [14]. The presence of soft tissue mass represents another important sign of malignancy; in a recent investigation about MRI findings in these tumors, the authors concluded that soft tissue mass was indicative of high-grade lesion whereas the presence of entrapped fat within the tumoral tissue was consistent with low-grade tumors [26]. Dynamic MRI was found useful in differential diagnosis between benign and malignant cartilaginous lesions; early uptake of gadolinium with an exponential curve was associated with malignant cartilaginous tumors [27]. Recently, the role of fluorodeoxyglucose positron emission tomography in differential diagnosis of cartilaginous tumors was investigated: the standard uptake value resulted to be correlated to the grade of malignancy, with a cutoff value between benign and malignant lesions ranging from 2 to 2.3 in two different reports, with statistical significance in only one of them [28, 29].

It is commonly accepted that the diagnostic approach to central low-grade CS must be multidisciplinary, requiring a trained and experienced team of radiologists, pathologists, oncologists, and surgeons.

In our opinion, small (<5 cm), asymptomatic, intraosseous cartilaginous lesions of long bones with absence of radiological and MRI signs of local aggressiveness should be observed as benign enchondroma, and no surgery or further investigations other than serial radiological follow-up are indicated.

In larger and/or symptomatic lesions, dynamic MRI or PET scan is indicated. If the adopted examination is negative, conservative monitoring of the lesion is the treatment of choice, unless the site and/or size of the lesion warrant surgical intervention with intralesional curettage and filling. If dynamic MRI or PET scan (SUV >2) is positive, a diagnosis of low-grade chondrosarcoma is to be assumed and decision-making on surgical treatment should be done considering imaging aspects, because histologic diagnosis of cartilaginous tumors requires analysis of the entire specimen and a biopsy sample is not enough for grading (progression of malignancy may be present, and in the same lesion different areas of benign enchondroma and malignant chondrosarcoma may coexist).

Grade 1 CS of the appendicular skeleton with no aggressive imaging features can be treated with intralesional curettage and local adjuvants. However, when aggressive biologic behavior is evident on imaging, segmental resection following surgical principles of malignant bone tumors seems more appropriate.

In our protocol, biopsy was omitted in cartilaginous lesions without radiographic aggressive behavior, and definitive histologic diagnosis was made on the entire tissue obtained by curettage. When an aggressive imaging pattern was evident, biopsy was performed to confirm diagnosis before resection. Since definitive diagnosis is possible only after examination of the complete specimen, the chance of inadvertent curettage of grade 2 CS must be considered, but after secondary appropriate treatment with wide resection, no adverse influence on outcome was reported [30]. Our personal therapeutic opinion was validated by the results of the present study: in our series, by accurate selection of patients based on imaging criteria, no inadvertent curettage of grade 2 CS was observed. Nonetheless, this possibility should always be considered, and adequate surgical exposure and technique must always be adopted to make a second procedure of wide resection feasible.

After intralesional curettage, local adjuvants on the cavity walls can be used to decrease the risk of local recurrence, although no definitive data are available to support this choice. Phenol plus ethanol [13, 14, 16] and cryosurgery [14–16, 20–22, 31] have been used as local adjuvants in curettage of central low-grade CS, and no significant difference in local control was observed between these different techniques. From previous in vitro studies, phenol appeared to be ineffective on cartilaginous tissue and protective properties of chondroid matrix against the cytotoxic action of phenol had been postulated [32]. On the contrary, recent investigations demonstrated in vitro the cytotoxic effect of phenol and ethanol on chondrosarcoma cells at minimum concentration of 1.5 % and 42.5 %, respectively [33]. Cryosurgery may be performed with liquid nitrogen, directly poured into the cavity, or employing cryoprobes using liquid nitrogen or argon gas. Cryotherapy induces deep necrosis (7–12 mm), and prophylactic internal fixation was recommended to decrease the risk of pathologic fracture [34]. After curettage, the bone cavity may be filled by bone grafts (autologous or allogenic) [13, 14, 16, 21, 31] or methylmethacrylate [10, 13, 14, 16, 17, 20–22], or may be left empty [14, 15]. Bone cement is also considered a local adjuvant due to the necrotizing effect of the exothermic reaction on the cavity wall.

According to the above-mentioned criteria, in our series of 85 cases of low-grade chondrosarcomas, we could perform conservative surgery (curettage and filling) in 64 patients, with only two cases of local recurrence. The results of treatment in our series confirm that low-grade chondrosarcoma has favorable prognosis with low rate of local recurrence and low or null rate of distant metastasis. This is in accordance with data reported by other authors after both intralesional curettage and segmental resection (Table 1).

**Table 1:** Treatment and results for low-grade chondrosarcoma in recent literature

Author [Ref.]	No. of patients, sites	Diagnosis	Follow-up (months)	Surgical treatment (no. of patients)	Adjuvants (other than cement)	Filling of defect after curettage	Recurrence (%)	Distant metastasis (%)	Postop. fractures (%)	Grade switch after LR
Verdegaal et al. [31]	85 limbs	CS low gr	86 (2–169)	Curettage	Phenol	Allografts	5.9	0	2.3	0/5
Funovics et al. [11]	70 all sites	CS low gr	81 (6–317)	Resection (36), curettage (34)	None	Cement	Intrales: 17.9, margin: 14.3	1.4	NA	None
Andreou et al. [8]	56 low grade out of 115 all grade, all sites	CS gr 1	144 (60–288) (follow-up of entire series, 115 pts)	Curettage or resection	NA	NA	26.8	5.3	NA	At least 3, NA entire number
Donati et al. [14]	31 limbs	CS gr 1	157 (66–296)	Resection (16), curettage (15)	Phenol (9), liquid nitr (3), none (3)	Cement (5), allografts (3), autografts (1), Cem + Pr (2), none (4)	Resection: 0, curettage: 13.3	0	Resect: 0, curett: 6.7	0/2
Mohler et al. [22]	46 all sites	CS gr 1/enchondroma	47 (18–134)	Curettage	Liquid nitr + H_2_O_2_	Cement	4.3	0	6.5	0/2
Souna et al. [15]	15 limbs	CS gr 1	96 (60–132)	Curettage	Liquid nitrogen	None	0	0	6.7	–
Aarons et al. [16]	32 limbs	CS gr 1	55 (24–203)	Resection (15), curettage (17)	Phenol (6), liquid nitr (3), H_2_O_2_ (1), none (7)	Cement (10), allografts (7)	Resection: 6.7, curettage:5.9	0	Resect: 20, curett: 5.9	0/2
Hanna et al. [17]	39 limbs	CS gr 0.5/CS gr 1	61 (36–104)	Curettage	None	Cement	5	0	0	0/2
Van Der Geest et al. [21]	130 NA	CS gr 1/enchondroma	60 (24–119)	Curettage	Liquid nitrogen	Allografts or autografts (cement in 3 cases)	1.5	0	14	0/2
Streitburger et al. [10]	69 all sites	CS gr 1	78.5 (2–365)	Resection (60; wide marg. 43), curettage (9)	H_2_O_2_	Cement (6)	14.5	5.8	NA	3/10
Leerapun et al. [13]	70 limbs	CS gr 1	102 (2–273)	Resection (57), curettage (13)	Phenol	Bone graft (12), cement (1)	2.8	2.8	NA	1/2
Ahlmann et al. [20]	10 all sites	CS low gr	38.5 (24–60)	Curettage	Argon gas cryoprobes	Cement	0	0	0	–
Our series	80 limbs	CS gr 1	72 (24–206)	Resection (23), curettage (57)	Phenol (40)	Allografts (53), cement (3), bone substit (1)	Resection: 0, curettage: 3.5	0	Curett: 1.7	1/2

Cem + Pr: after performing curettage, removal of metaphysis together with the epiphysis, using the removed bone to obtain a cemented composite prosthesis

*gr* grade, *NA* not available data, *Intrales* intralesional margins, *Margin* marginal margins

Leerapun et al. [13], in a series of 70 patients treated with intralesional curettage in 13 cases and segmental resection in 57 cases, reported one local recurrence in each group of patients and no differences in survival. The authors concluded that intralesional curettage should be reserved for central grade 1 CS of long bones without aggressive imaging features such as soft tissue mass and cortical disruption. A similar conclusion was reached by Donati et al. [14], who reported 31 cases of central grade 1 CS of long bones with long-term follow-up (mean 157 months, minimum 66 months), treated with curettage in 15 cases and resection in 16 cases. Two local recurrences were observed in the curettage group, and bone enlargement and cortical thinning were imaging features strongly indicative of higher biological aggressiveness of the tumor. No difference in local recurrence rate between curettage and segmental resection of central grade 1 CS of long bones was reported also by Aarons et al. [16], and lower complication rate and better functional outcome were observed after intralesional surgery.

According to these data, the complication rate in our series is consistently higher after segmental resection (28.6 %) than after intralesional curettage (1.6 %). Local recurrence occurred only after curettage in our series, but with a very low rate (3.1 % after curettage, 0 % after resection).

Our study has several limitations. First, it is a retrospective analysis and not a prospective study, but the rarity of this pathology and the need for long oncological follow-up make it difficult to accomplish a prospective study. Second, comparison of different treatments (curettage versus wide resection) was not randomized, but on the basis of current knowledge about this pathology it is not possible to accomplish a randomized study as different presentations of the disease suggest specific surgical choices to avoid undertreatment of locally aggressive tumors or overtreatment of small and less aggressive lesions. Third, the follow-up of our patients is significant, but still too short for definitive considerations about oncological results.

With the above-mentioned limitations, our experience confirms that grade 1 chondrosarcoma of the appendicular skeleton can usually be treated with intralesional curettage and local adjuvants. Only when aggressive biologic behavior is evident on imaging (cortical relevant thinning, enlargement, and/or breakthrough) should wide resection following surgical principles of malignant bone tumors be considered.

Considering the very low rate of distant metastases in all reported series, appropriate oncological follow-up should be defined to avoid excessive exposure to several CT scans of the chest, as routinely performed in bone sarcoma.
